# Introduction of Mouse Embryonic Fibroblasts into Early Embryos: From Confusion to Constructive Discussion. Comment on Savatier, P. Introduction of Mouse Embryonic Fibroblasts into Early Embryos Causes Reprogramming and (Con)fusion. *Cells* 2021, *10*, 772

**DOI:** 10.3390/cells10061534

**Published:** 2021-06-18

**Authors:** Krystyna Żyżyńska-Galeńska, Jolanta Karasiewicz, Agnieszka Bernat

**Affiliations:** 1 Department of Experimental Embryology, Institute of Genetics and Animal Biotechnology, Polish Academy of Sciences, Jastrzębiec, 05-552 Magdalenka, Poland; k.zyzynska@igbzpan.pl (K.Ż.-G.); jolantakarasiewicz@op.pl (J.K.); 2 Laboratory of Molecular Diagnostics, Department of Biotechnology, Intercollegiate Faculty of Biotechnology, University of Gdańsk & Medical University of Gdańsk, 80-307 Gdańsk, Poland

We would like to address the issues raised by Pierre Savatier in “Introduction of Mouse Embryonic Fibroblasts into Early Embryos Causes Reprogramming and (Con)Fusion” [1] regarding our article recently published in Cells [2], with hope to dispel the confusion by developing the discussion further.

*“To ascertain the pluripotency of the reprogrammed MEFs, studies must still address whether these cells express markers of ectoderm, mesoderm, and definitive endoderm.”*

Although we did not show proof that injected cells differentiate terminally into three germ layers derivatives, we showed that introduced MEFs integrated with the embryo, reprogrammed at the blastocyst stage and survived in foetuses, foetal membranes and adult tissues. The low percentage of cells found in foetuses would generate difficulties in the visual tracking of cell progenies in fetal tissue slices, and it is highly possible that finding marked cells would be elusive. We take the position that flow cytometry is a preferable method for detecting such low numbers of cells in tissues. 

Additionally, we can speculate that, if injected, MEFs would not undergo reprogramming in early embryos, meaning that most probably cells undergo senescence and eventually die off, as it takes place when MEFs are cultured in vitro. On the other hand, if single cells can survive in the developing embryo without senescence and without reprogramming, stalled in their state and not eliminated from rapidly growing foetuses, their number would be extremely low (below the detection threshold), as only three to four cells are introduced into the embryo. 

Thus, the presence of donor DNA in the brain and heart cells of adult mice are evidence of the ectodermal and mesodermal lineages, respectively, of those cells.

We do not agree that injected MEFs have been mis-specified in the embryo during the process of reprogramming. The cells expressing both NANOG and GATA4 were not classified as either epiblast or primitive endoderm cells, but were described as a separate group (see Figure 2F, Table S1 in the original paper [2]). It is worth mentioning that in the inner cell mass of native blastocysts some cells still co-expressed both markers even until the stage of a total of 80 cells [3].

*“Notably, 71% of the foetuses that developed from embryos with confirmed MEF contribution were growth-retarded, abnormal, or resorbed.”*

The embryos for DNA analysis were selected to check the possibility of whether donor cells were present more often in retarded, delayed, abnormal or normal embryos. These data—quoted in the Editorial—are shown in Table S4 in original paper. The frequency of normal development in all injected embryos is shown in Table S2 [2]. As mentioned in the text: since normal development was very high at day E13.5 (82.4%), we went to the earlier stage E10.5–12.5 to collect the developmental failures. Still, in the latter group, 28.9% were normal embryos.

*“Hybrid tetraploid cells were detected in virtually all fetal and adult tissue samples, with rates ranging from 0.7% to 25% of all MEF-originating cells. The results are unclear regarding whether these tetraploid cells resulted from the persistence of the MEF-derived hybrid cells identified in the blastocysts as previously shown or from de novo fusions taking place during organogenesis.”*

In our earlier work [4] we found the hybrid isozyme of glucose phosphate isomerase (GPI-1AB) in 32.4% of E11–E14 embryos (Table 4 therein). Hybrid isozyme can only appear when donor GPI-1A and recipient GPI-1B are present in the same cell. We considered this the prove that donor/recipient hybrid (tetraploid) cells were derived from cell fusion before organogenesis.

*“Hybrid donor/host cells have not been systematically characterized for the expression of lineage markers, leaving their identity uncertain. Particularly, the results are unclear regarding whether these hybrid donor/host cells express pluripotency or lineage markers that correspond to their spatial allocation”.*

Hybrid donor/host cells originating from cell fusion are tetraploid, and as such they are less interesting than diploid reprogrammed cells both for studying mechanisms of cell interactions in development, and in testing human PSC in interspecific chimeras. This is why they attained less attention in our paper. However, it is not a question of whether the results were unclear or uncertain. Simply, the claimed properties are unknown.

*“MEFs are introduced inside the morula, a procedure that inevitably leads to blastomere damage.”*

The procedure of introducing cells into the morula does not inevitably lead to the blastomere puncture. When approaching the center of the embryo, the smooth pipette delicately pushes the blastomeres aside. In order to verify the suggested hypothesis that puncturing blastomeres would be the driving force of MEF reprogramming, we have analyzed photos of 165 injected embryos (from different experimental setups). We verified the presence of punctured blastomere(s) and combined it with the reprogrammed fate of injected cells (Figure 1A–C and Table 1). In 165 embryos analyzed, we confirmed the destruction of blastomeres in 86 embryos (52%), but only in eight blastocysts were the reprogrammed positive cells (NANOG, GATA4 or CDX2) present (Table 1). In 63 embryos, no punctured blastomeres were identified and there were 18 blastocysts with reprogrammed cells. In 58 blastocysts out of the analyzed 165, a punctured blastomere together with MEF was eliminated from embryo (Figure 1A).

These results indicate that the “punctured blastomere” theory cannot account for most of reprogramming events, and other mechanisms must be implicated.

It is possible, however, that micropunctures and micromechanical damage are caused during the procedure. Therefore, studying the re-establishing of physical connections inside morula after MEFs are placed in between blastomeres would be promising in the search for the reprogramming trigger.

*“…cell microinjection destroys one or more blastomeres in the host embryos, resulting in the release of cytoplasmic determinants in the embryonic environment and triggering reprogramming in a process comparable with SCNT.”*

If a blastomere is destroyed, its content would be released into the intercellular space. Blastomere content would only reach the cytoplasm of a fibroblast were that cell membrane of the fibroblast destroyed. That was not observed. Therefore *“triggering reprogramming in the process comparable with SCNT”* is simply not possible, as the contact of injected cell nucleus and recipient cytoplasm is required for reprogramming by somatic cell nuclear transfer.

One could consider the reprogramming by cytoplasmic extracts [5,6,7]. However, to achieve this, a procedure of permeabilization of recipient cell membrane would need to be performed. Again, the integral fibroblast cell membrane prevents the penetration of released blastomere’s content. Thus, we can exclude also that possibility from potential mechanisms of somatic cell reprogramming in morula.

In our opinion, when reprogramming in an embryonic niche is applied for interspecies chimera formation, a number of factors have to be taken into consideration, adding the further load to the process, which is complex per se.

The window of MEF susceptibility to reprogramming in an embryonic niche of early morula can be very narrow. MEF cell cultures are heterologous in nature, as all primary cell cultures are, and differences in reprogramming capacity may arise from differences between single particular cells. The transcriptional profiling and comparison of primed and naïve mouse ESCs/iPSCs and MEFs could help to elucidate whether there are similarities that could explain the phenomenon. Interestingly, although we did not observe the expression of Oct4 and Nanog pluripotency factors in MEFs, we could clearly see high expression of KLF4 in MEF (Figure 1D). This is particularly interesting, as KLF4 is one of the transcription factors involved in the regulation of naïve pluripotency, and clearly the limiting factor in transition from primed to naïve pluripotency. Indeed, KLF4 overexpression has been shown to reprogram EpiSc to a naïve state [8], and its forced expression with KLF2 has been tested as a strategy to boost pluripotency in primed rabbit ESC [9].

None of the experiments aimed at producing chimera with primed, naïve-like or naïve pluripotent cells attempted the injection of cells in between blastomeres of early morula. The majority of the works were performed by injecting cells into blastocyst once cell fates (ICM/TE) had already been established. In light of above, injecting the cells into more plastic and unsealed environments would surely be an appealing approach when the reprogramming of cells is necessary, in order to procure the colonization of latter inner cell mass.

It is also worth mentioning that at the time of epiblast-primitive endoderm specification, epiblast precursors exhibit less plasticity than precursors of primitive endoderm (PrE). This phenomenon is explained by the differences in responsiveness to extracellular signaling in these cells. Most probably, the early embryo environment restricts the fate choice of epiblast, but not PrE precursors, to ensure preservation of pluripotent foetal lineage [10].

*“Arguably, the findings of this paper shed new light on interspecies chimeras, systemic chimeras produced by introducing the embryo-derived or induced pluripotent stem cells (PSC) of one species, usually human or rhesus monkey, into the preimplantation embryos of a different species, typically mouse, rabbit or pig.”*

Before producing “*systemic chimeras*” some questions might be asked. Starting from the genetic background of the recipient, to the developmental asynchrony between donor and recipient, the knowledge on recipient morula development stages and the corresponding window of injection time, through tetraploid contribution of recipient or donor cells, and up to the contribution of hybrid MEF/ES cells to chimeras.

The above set of areas could be a formidable source of inspiration for new scientific questions.

## Figures and Tables

**Figure 1:**
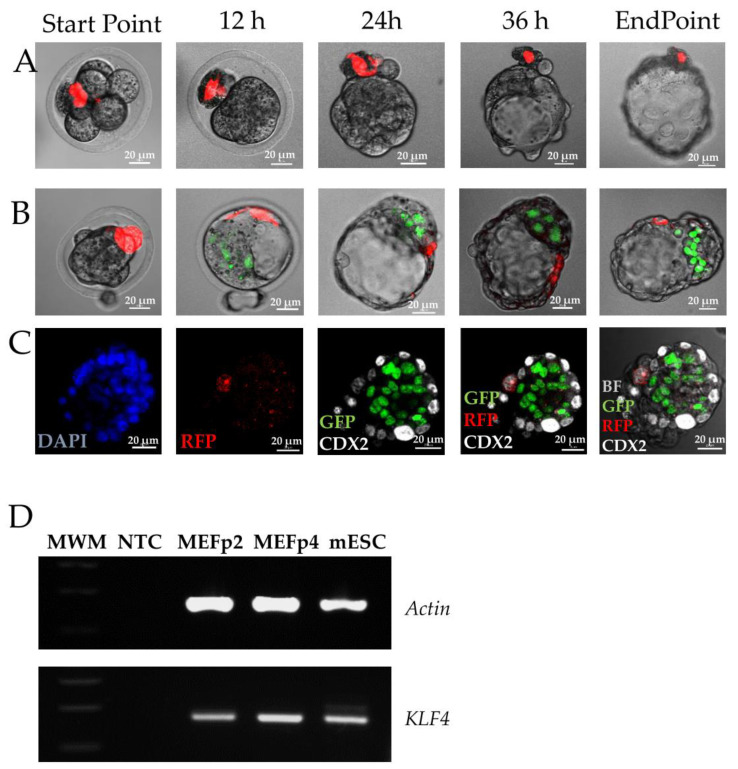
Development to the blastocyst of chimeric embryos carrying MEF cell-expressing RFP (red). (**A**) Chimeric embryo with MEF cell-expressing RFP (red) being eliminated from the embryo after blastomere puncturing; (**B**) development of chimeric embryo to blastocyst with MEF cell-expressing RFP (red), integrated with the embryo without blastomere puncturing; (**C**) immunofluorescent staining of blastocyst with MEF cell expressing RFP (red), integrated with the embryo without blastomere puncturing; (**D**) RT-PCR analysis of KLF4 expression in MEF cells at passage 2 (p2) and 4 (p4), as compared to mouse embryonic stem cells. Materials and methods are described in [2].

**Table 1:** The presence of reprogrammed MEF cells in blastocysts with and without destroyed blastomeres.

Number of Blastocysts	With Destroyed Blastomeres	Without Destroyed Blastomeres	Difficult to Determine	Total
Total	86	63	16	165
With reprogrammed MEFs	8 (9.3%)	18 (28.6%)	1	27

## Data Availability

The data presented in this study are available on request from the corresponding author.

## References

[1] Savatier P. (2021). Introduction of Mouse Embryonic Fibroblasts into Early Embryos Causes Reprogramming and (Con)fusion. Cells.

[2] Żyżyńska-Galeńska K., Bernat A., Piliszek A., Karasiewicz J., Szablisty E., Sacharczuk M., Brewińska-Olchowik M., Bochenek M., Grabarek J., Modliński J.A. (2021). Embryonic Environmental Niche Reprograms Somatic Cells to Express Pluripotency Markers and Participate in Adult Chimaeras. Cells.

[3] Plusa B., Piliszek A., Frankenberg S., Artus J., Hadjantonakis A.-K. (2008). Distinct Sequential Cell Behaviours Direct Primitive Endoderm Formation in the Mouse Blastocyst. Development.

[4] Piliszek A., Modliński J.A., Pyśniak K., Karasiewicz J. (2007). Foetal Fibroblasts Introduced to Cleaving Mouse Embryos Contribute to Full-Term Development. Reproduction.

[5] Håkelien A.-M., Landsverk H.B., Robl J.M., Skålhegg B.S., Collas P. (2002). Reprogramming Fibroblasts to Express T-Cell Functions Using Cell Extracts. Nat. Biotechnol..

[6] Håkelien A.-M., Collas P. (2002). Novel Approaches to Transdifferentiation. Cloning Stem Cells.

[7] Collas P., Taranger C.K. (2006). Epigenetic Reprogramming of Nuclei Using Cell Extracts. Stem Cell Rev..

[8] Guo G., Yang J., Nichols J., Hall J.S., Eyres I., Mansfield W., Smith A. (2009). Klf4 Reverts Developmentally Programmed Restriction of Ground State Pluripotency. Development.

[9] Tapponnier Y., Afanassieff M., Aksoy I., Aubry M., Moulin A., Medjani L., Bouchereau W., Mayère C., Osteil P., Nurse-Francis J. (2017). Reprogramming of Rabbit Induced Pluripotent Stem Cells toward Epiblast and Chimeric Competency Using Krüppel-like Factors. Stem Cell Res..

[10] Grabarek J.B., Zyzynska K., Saiz N., Piliszek A., Frankenberg S., Nichols J., Hadjantonakis A.-K., Plusa B. (2012). Differential Plasticity of Epiblast and Primitive Endoderm Precursors within the ICM of the Early Mouse Embryo. Development.

